# The Cardio- and Neuroprotective Effects of Corvitin and 2-Oxoglutarate in Rats with Pituitrin-Isoproterenol-Induced Myocardial Damage

**DOI:** 10.1155/2018/9302414

**Published:** 2018-09-03

**Authors:** V. Tkachenko, Y. Kovalchuk, N. Bondarenko, О. Bondarenko, G. Ushakova, A. Shevtsova

**Affiliations:** ^1^SI “Dnipropetrovsk Medical Academy of the Ministry of Health of Ukraine”, Dnipro, Ukraine; ^2^Oles Honchar Dnipro National University, Dnipro, Ukraine

## Abstract

Heart diseases, especially acute coronary syndrome (ACS), are among the most severe illnesses that often lead to death. Despite significant advances in the prevention and treatment of ACS, the incidence of the disease and its complications are very serious. The imbalance between pro- and antioxidant systems, the formation of active carbonyl compounds, and the end products of glycation in the blood and tissues are the key moments in the development of heart and neurological disorders leading to a change of behavioral responses. So, the search for antioxidants with cardio- and neuroprotective effects is an urgent task. This study was aimed at evaluating the effects of Corvitin and 2-oxoglutarate on physiological parameters, heart histology, and markers of carbonyl/oxidative stress of rats with pituitrin-isoproterenol-induced myocardial damage (PIMD). Increased sweating, tachycardia, significantly decreased locomotor and exploratory activity, changes of ECG, heart histology, and biochemical changes were observed in the PIMD-group. The administration of Corvitin or 2-OG led to the recovery of locomotor and cognitive activities of the rats, improvement in heart histology, a decrease in the levels of thiobarbituric acid reactive substances, advanced glycated end products, and various changes in the activity of the antioxidant enzymes, 6 days after PIMD. So, Corvitin and exogenous 2-OG show cardio- and neuroprotective effects through the decrease of carbonyl/oxidative stress and regulation of the activity of the antioxidant system.

## 1. Introduction

Cardiovascular diseases (CVDs), notably, acute coronary syndromes (ACS), are among the most severe illnesses that often lead to death. According to the forecast of the World Health Organization, these diseases are projected to dominate as the primary cause of death by 2020 [1]. Despite significant advances in the prevention and treatment of CVD, the incidence of the diseases and their complications remain high. According to unofficial statistical data, more than 580 thousand people died for various reasons throughout 2016-2017 in Ukraine, with one-third of them dying from heart and blood vessel diseases [2, 3]. Thus, the search for drugs that have metabolically grounded cardioprotective properties is topical.

Among the well-known factors that trigger the development of coronary heart disease are discussed reactive oxygen species (ROS) and other free radicals, which attack lipids containing unsaturated fatty acids, mediating the chain of reactions of lipid peroxidation [4, 5]. Processes of lipid peroxidation (LPO) lead to disruption of the structure and permeability of cardiomyocyte membranes and to the changes of their metabolism [6]. Furthermore, LPO plays an important role in atherogenesis through oxidation of low-density lipoprotein phospholipids, which have proinflammatory and proatherogenic properties [7]. Intense of lipid peroxidation and the degree of cell damage have been shown to be highly dependent on the activity of antioxidant enzymes. The imbalance between pro- and antioxidant systems in cells of the heart muscle and vascular endothelium is the crucial point of oxidative stress and is accompanied by the activation of signaling pathways responsible for gene expression of proinflammatory and proapoptotic proteins, creating conditions for the development of acute myocardial infarction [8, 9]. Oxidative stress is also associated with the appearance of active carbonyl compounds, which modify proteins, nucleic acids, and other amino compounds with formation of advanced glycation end products (AGEs) [10]. Traditionally, the process of nonenzymatic glycation was associated with prolonged diabetic hyperglycemia. Advanced glycation end products are linked to the development of various complications of diabetes, including cardiorenal syndrome [11], but the role of the AGEs in nondiabetic heart damages is unclear. The evaluation of oxidative and carbonyl stress markers under experimental myocardial ischemia can provide additional information on the molecular mechanisms of development of CVD.

Epidemiological studies have shown that 30% to 50% of acute myocardial infarctions are the results of an emotional provocation [12], which leads to changes in the behavior of the patients. These changes are not only pain inflicted but are also caused by the damage of stellate ganglions, as well as by the dysfunction of ionic channels of the brain and heart [13, 14]. The extensive oxidative metabolism due to ischemic attack is accompanied by a concomitant generation of high amounts of reactive oxygen, nitrogen, and carbonyl species in the brain [15] So, the study of the behavioral reactions under conditions of myocardial damage are potentially useful in predicting possible neuronal complications, in monitoring the efficacy, and in choosing the more rational therapy. The application of a universally accepted medical treatment including *β*-blockers, anticoagulants, antithrombotic agents, and nitrates in some cases gives no anticipated results. Several antioxidants have been tested for their possible cardio- and neuroprotective actions against hypoxia-induced diseases; bioflavonoid quercetin is the most popular among them. Quercetin is used for prevention and treatment of cardiovascular and neurological disorders [16–18] owing to its ability to inhibit ROS production and the activity of oxidative enzymes (lipoxygenase and xanthine oxidase), suppress inflammatory processes, and regulate the content of nitric oxide [19, 20]. However, poor intestinal solubility and absorption, as well as rapid neutralization after oral administration—the “flavonoid paradox” [21, 22]—limit the bioavailability of this bioflavonoid. Currently, Corvitin—a water-soluble form of quercetin for intravenous injections—is used as a drug with a pronounced antiischemic, antistroke, and antiinfarction activity [23, 24]. Corvitin has a high bioavailability with a sufficiently low level of toxicity [25]. It has been shown that cardioprotective properties of Corvitin could be attributed to its ability to alter the activity of proteolytic enzymes [26, 27], but the effect of this drug on the state of the heart muscle and the markers of carbonyl-oxidative stress have not been adequately studied.

Another group of cardioprotective medications is based on their ability to improve the energy metabolism in a postischemic heart [28, 29]. 2-Oxoglutarate (2-OG, also known as alpha-ketoglutarate) is among these substances. As a central metabolite of the Krebs cycle, it contributes to the regulation of anabolic and catabolic reactions of the TCA products and substrates, thereby regulating amino acid synthesis, ATP production, and reducing equivalent (NAD^+^/NADH) generation, which in turn can influence ROS levels [30, 31]. Previous data suggest that in different cases of induced oxidative stress *in vitro* or *in vivo*, 2-OG stabilizes redox homeostasis and improves arterial elasticity in aged mice [32]. However, the impacts of this substance on the morphology and metabolism of the heart, as well as on redox balance, after an ischemic attack is poorly understood. The purpose of this work was to evaluate the effect of Corvitin and 2-oxoglutarate on physiological parameters, behavioral reactions, the activity of antioxidative enzymes, and the content of advanced glycated products under experimental myocardial damage in rats.

## 2. Materials and Methods

### 2.1. Animals

The care and use of animals was conducted in compliance with the principles outlined in the *Guide of the Care and Use of Experimental Animals* in accordance with the ethical standards established by the Ukrainian law no. 3447-IV, dated 21.02.06 “On the protection of animals from cruelty,” and it was approved by the Local Ethics Review Committee on Animal Experiments in Dnipropetrovsk Medical Academy (Dnipro, Ukraine). Wistar male rats weighing 195 ± 50 g were exposed to standard conditions, such as reverse 12-hour light-dark cycle (light 07:00–19:00) and provided with laboratory nutrition and water *ad libitum*.

### 2.2. Reagents

All the reagents used in this study were clean and chemically pure: sodium thiopental (ARTERIUM, Ukraine); Corvitin (quercetin for intravenous injection and in granules, Borshchahivskiy Chemical-Pharmaceutical Plant, CJSC, Kiev, Ukraine); 2-oxoglutarate (SGPlus, Malmo, Sweden); pituitrin (Endokrininiai, Lithuania); isoproterenol (isoprenaline hydrochloride); alpha-amylase (A3176), oxidized glutathione, 5,5′-dithiobis-2-nitrobenzoic acid, tetramethylenediamine, and bovine serum albumin were the products from Sigma-Aldrich (St. Louis, MO, USA); quinine hydrochloride (Buchler GmbH, Germany); thiobarbituric acid (Kharkovreаchim, Ukraine); reduced glutathione (AppliChem GmbH, Germany); *β*-nicotinamide adenine dinucleotide 2′-phosphate reduced tetrasodium salt hydrate (Oriental yeast co., Ltd, Japan); Coomassie G250 (SERVA, Germany); test system for determination of glucose and test system for determining hemoglobin concentration were the products of Phyllis-Diagnosis (Ukraine). All other chemicals and solvents were at analytical grade level.

### 2.3. Myocardial Damage Model

The rat model of isoproterenol-induced myocardial injury serves as a well-accepted standardized model for the evaluation of several cardiac dysfunctions and for studying the efficacy of various natural and synthetic cardioprotective agents [33–35]. We used the model proposed by Belenichev and others [36], which follows the principle that the imbalance between the supply and the metabolic demand of oxygen by the heart muscle is achieved by the combined administration of pituitrin and isoproterenol. The drugs were administered as follows: first, pituitrin (0.5 U/kg)—intraperitoneally, followed by isoproterenol after 20 minutes (100 mg/kg)—subcutaneously. The same injections of pituitrin and isoproterenol were repeated 6 h and 24 h later.

### 2.4. Experimental Protocol

Animals were randomly divided into four groups, ten rats in each group. Group 1 (control animals) received saline injections (1 ml/kg) for 5 days; group 2 animals underwent pituitrin-isoproterenol myocardial damage (PIMD); group 3 rats received intra-abdominally the Corvitin (42 mg/kg) for 5 days after PIMD onset (day 1: three doses at intervals 1, 2, and 12 hours; days 2 and 3: two doses within 12 hours of each; and days 4 and 5: one dose 24 hours apart). The animals were removed from the experiment a day after the last injection of Corvitin. Group 4 animals received 1% solution of 2-oxoglutarate in drinking water *ad libitum* for 6 days after PIМD onset. Each animal consumed in average 5-6 ml of 2-OG solution per day.

### 2.5. Monitoring Physiological Parameters of the Experimental Animals

The physiological state (weight, respiration, motor activity, and heart rate) of the animals was under constant control during the experiment. The heart functioning was evaluated using ECG recordings and analyzed with the computer cardio complex, CardioLab 2000 (Ukraine). Behavioral responses of rats were examined with the Buresh open-field test [37], using a white painted plywood floor (80 × 80 cm) divided by black lines into 16 squares (20 × 20 cm) and elevated 50 cm above the ground. Holes (*n*=9) were situated at the intersection of the four internal squares (*d* = 4 cm). Rats were placed in one corner and directed to the center. Videos of their behaviors were recorded for 3 minutes. To prevent the rats from being distracted, the field surface was treated with 70% ethanol solution after each animal test. Evaluations were made for the number of lines crossed, vertical stands, hole-looking, and defecation by each animal. This approach allows for simultaneous exploration of locomotion, exploratory activity, and the autonomic-emotional state of animals. This test was carried out in the light cycle phase at the beginning of the experiment, 24 hours after PIMD, and on the 6th day of 2-OG and Corvitin administration.

### 2.6. Histopathological Studies

At the end of the experiment, the animals were sacrificed using sodium thiopental (40 *µ*g/kg) and decapitated following the ethical standards. Rat hearts were harvested and fixed in 10% formalin buffered in PBS (pH = 7.4) for 24 hours at room temperature, dehydrated in increasing concentrations of isopropanol, cleared in xylene, and embedded in paraffin. At least four 5 *μ*m thick slices were obtained from each specimen for the histological study of the structural changes in the left ventricle myocardium. Two series of sections were then stained with Periodic Acid Schiff Staining Technique (PAS) [38] for glycogen detection: one set was preincubated with diastase and then stained, while the other was stained without diastase pretreatment. All sections were evaluated repeatedly by two independent observers using the light microscopy. Two characteristics for histological evaluation identified as the most common for all groups were used: inflammatory infiltration and PAS-staining intensity of cardiomyocytes. All histological features were assessed in nine different fields of view per specimen, at a magnification of ×400. The areas of view were chosen for the maximal coverage of subepicardial, intramural, and subendocardial layers of rat myocardium, thus aggregating three fields of observation for one segment.

### 2.7. Biochemical Analysis

The plasma and red blood cells of experimental animals were used for biochemical analysis. Lipid peroxidation was estimated by measuring levels of thiobarbituric acid reactive substances (TBARS) in plasma by the spectrophotometric method [39]. The activity of the antioxidant enzymes, such as superoxide dismutase (SOD), catalase, glutathione peroxidase (GP), and glutathione reductase (GR), was evaluated in hemolyzed erythrocytes. The content of SOD was determined by calculating the speed of auto-oxidation of quercetin in the presence of tetramethylethylenediamine [40]; the activity of catalase was measured by the reaction with ammonium molybdate [41]; the activity of GP was deduced from the reaction between sulfhydryl groups of reduced glutathione and Elman's reagent [42]; and the GR-activity was assessed by the NADPH-dependent conversion of oxidized glutathione to its reduced form [43]. The total protein content was determined by the Bradford microassay [44].

### 2.8. AGEs

The levels of advanced glycated end products (AGEs) were measured by quantitative fluorescence [45], using Hoefer DQ 2000 Fluorometer (USA) with fixed wavelengths (excitation/emission = 365 nm/460 nm). The fluorescent emission of plasma samples (10-fold diluted in 0.9% sodium saline) was measured at room temperature in a 1 cm quartz cuvette. The measurement results were expressed in arbitrary units (AU) using quinine hydrochloride (60 mg/l) as a standard solution, the fluorescence of which was taken as 1000 AU. The results of the measuring were comprised with fluorescence of glycated albumin; their ratio was calculated and used for evaluation of the AGEs content.

### 2.9. Statistical Analysis

Оbtained data are represented as mean ± standard deviation of three independent determinations, using Statistica 6.0 Software, Inc. Statistical analysis was performed by the unpaired two-tailed Student's *t*-test and one-way analysis of variance (ANOVA). Values with *P* < 0.05 were considered statistically significant.

## 3. Results

### 3.1. Effects of Corvitin and 2-Oxoglutarate on Physiological Indices of PIMD-Rats

**Figure 1:**
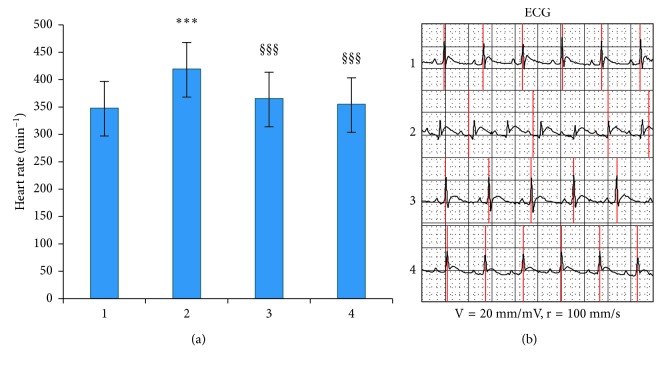
Effects of Corvitin and 2-oxoglutarate on heart rate (a) and ECG (b) in experimental groups. 1, control group; 2, rats with pituitrin-isoproterenol myocardial damage (PIMD); 3, rats with PIMD + Corvitin; 4, rats with PIMD + 2-OG; ^*∗∗∗*^*P* < 0.001, compared to control; ^$$$^*P* < 0.001, compared to PIMD-group.

Injections of isoproterenol and pituitrin led to the deterioration of the physical condition of experimental rats: polyuria, increased sweating, lethargy, and tachycardia were observed. The weight of control and experimental animals did not differ during the experiment −200 ± 20 g. The heart rate (HR) was elevated on 20% in the PIMD-rats, and its value significant decreased after the treatment by Corvitin (on 13%) and 2-OG (on 15%) in comparison with untreated animals (*P* < 0.001, Figure 1(a)). Substantial changes in the ECG configuration occurred on the 6th day after the pituitrin-isoproterenol injections. The detected changes were typical for ischemic myocardial injury; reduction of R wave amplitude to 0.46 ± 0.01 mV (0.56 ± 0.01 mV in control); and elevation of the ST segment to 1.81 ± 0.10 mm and its extension relative to the baseline. These changes indicate damage to the anterior wall of the left ventricle and disturbances of repolarization. Improvements in the ECG configuration were noted in rats of the 3rd group after the administration of Corvitin; the R wave amplitude increased to values like those of the control group, and there was no ST-segment elevation. A moderate amelioration of ECG was observed in the group of animals treated with 2-OG. An R wave amplitude recovery and a decreased ST segment were detected, compared to the second group, although the elevation of the S wave remained at 1.32 ± 0.01 mm (Figure 1(b)).

### 3.2. Effects of Corvitin and 2-Oxoglutarate on the Behavior of PIMD-Rats

**Figure 2:**
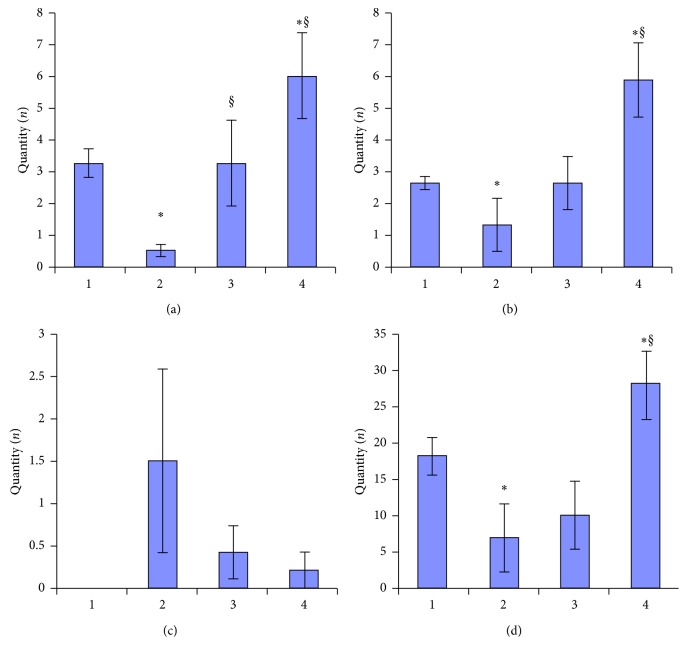
Influence of Corvitin and 2-oxoglutarate on behavioral activity in experimental groups: (a) vertical rack; (b) hole-peeping; (c) vegetative stress; (d) intersection of squares lines. 1, control group; 2, rats with PIMD; 3, rats with PIMD + Corvitin; 4, rats with PIMD +2-OG; ^*∗*^*P* < 0.05, compared to control; §*P* < 0.05, compared to PIMD-group.

Results of the open-field test showed changes in the behavioral activity of animals after pituitrin-isoproterenol-induced heart damage. The locomotor and exploratory activity (intersecting square lines, hole-looking, and the number of vertical racks) significantly decreased. On the contrary, the number of boluses increased in this group of rats compared to the control group. The administration of Corvitin led to the recovery of locomotor and cognitive activities of the experimental rats. The number of line-crossing increased, as did the frequency of hole-looking, and the number of vertical stands of the animals of this group. The administration of 2-OG after the acquired myocardial damage also led to a recovery in the animal exploratory activity (vertical rack and hole-looking) and reduced vegetative stress. Moreover, the animals were more active (Figure 2).

### 3.3. Effects of Corvitin and 2-Oxoglutarate on Histopathologic Changes Associated with PIMD in Rats

**Figure 3:**
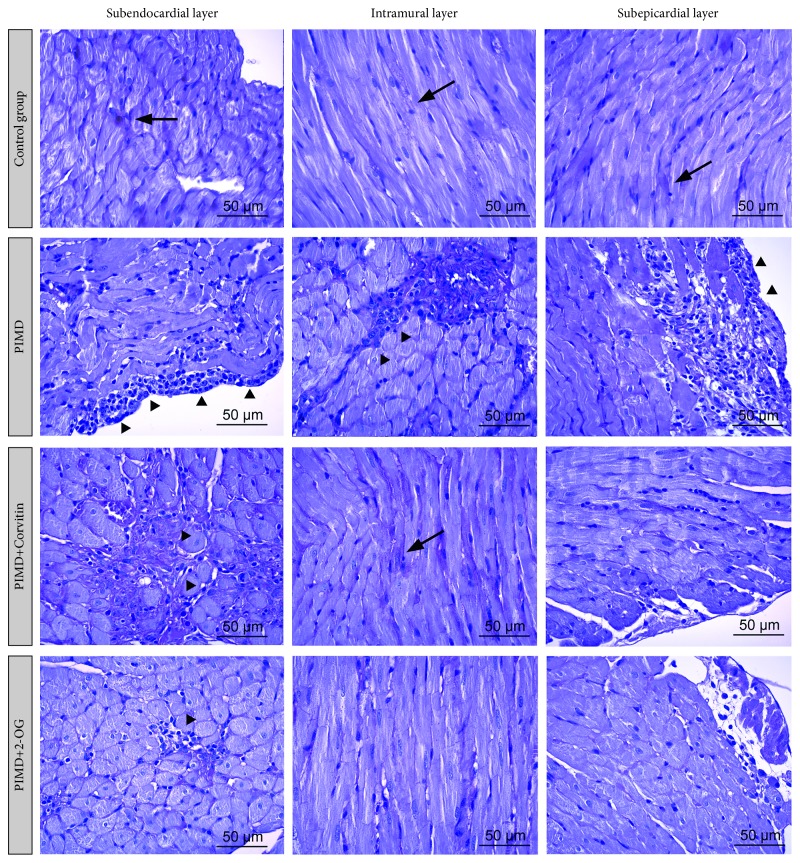
Histological comparison of the left ventricle myocardium in investigated groups. Arrows: prominent glycogen deposits; arrowheads: inflammatory infiltrate. PAS staining (magnification, 400x).

We found almost identical histopathological changes in samples of the left ventricular myocardium of all groups with PIMD, which differed only in their severity and distribution within the heart. These stereotypic changes comprised necrotic cardiomyocytes and inflammatory infiltrates of perivascular and interstitial localizations containing mostly lymphocytes, histiocytes, monocytes, and some plasma cells. The minimal signs of interstitial fibrosis were also noted. Additionally, the hyperemia of the capillary bed and the separation of the muscle tissue into bundles with swelled-up individual fibers were observed in some samples of these groups. Vacuolated cytoplasm and condensed dark-colored nuclei represented necrotic patterns in cardiomyocytes. Staining by PAS-technik displayed an apparent depletion of glycogen around heart attack-like lesions. It is important to note that the distribution of these changes varied in the studied groups in subendocardial, intramuscular, and subepicardial layers of the myocardium (Figure 3).

Microscopic comparisons of left ventricles of rats of the PIMD-group without Corvitin or 2-OG therapy revealed that postischemic and postnecrotic alterations comprised the highest depletion of glycogen in the sarcoplasm of cardiomyocytes and the most severe inflammatory infiltrates, located predominantly in the subepicardial layer. Perivascular edema and interstitial fibrosis were recognized in all myocardial segments of this group. The animals with PIMD demonstrated that intramural formation of multiple small foci tended to merge and consisted of lymphocyte-macrophage infiltrates with single polymorphonuclear leukocytes on the 6th day of follow-up. Besides focal infiltrates, a diffused mononuclear infiltration of myocardial stroma was observed, and the foci of necrotized cardiomyocytes were found predominantly in the subepicardial layer.

Animals of the third investigated group that received Corvitin after PIMD-induction presented less prominent inflammatory changes in the myocardium; the number of lymphocyte-macrophage infiltrates decreased, mostly located at the subendocardial and subepicardial levels. The foci of necrosis and myofibril destruction were randomly characterized, and the manifestations of perivascular edema and edema of the vascular wall also seemed insignificant. Glycogen-rich areas were predominantly observed in the intramural layer of the myocardium. The administration of 2-OG, for 6 days, to PIMD-rats was characterized by a significant decrease in inflammatory response and by a mild increase in the glycogen content in cardiomyocytes. The inflammation in the myocardium of these animals was the least pronounced compared with the previously described groups; it was spread all over the place. The changes in the microvasculature were comparable to those in the second group. The number of glycogen-rich cardiac myocytes in that group was relatively higher than that in the PIMD-group, but it was significantly less than the number in control and in PIMD + Corvitin groups.

### 3.4. Effects of Corvitin and 2-Oxoglutarate on Indices of Carbonyl/Oxidative Stress and Antioxidant Enzymes

**Figure 4:**
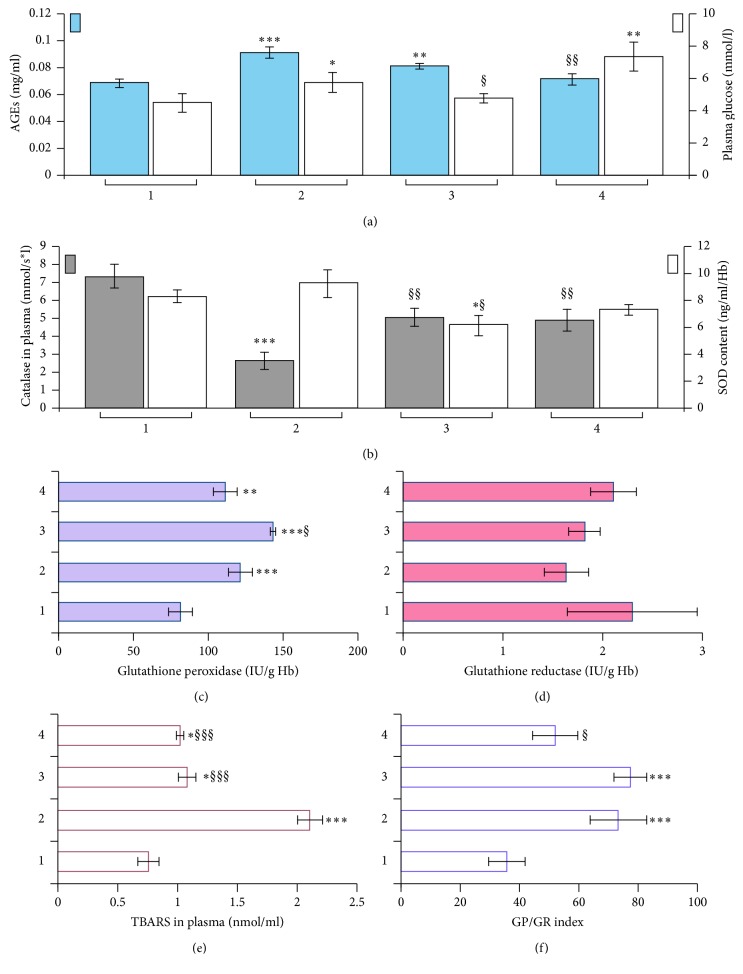
Influence of Corvitin and 2-oxoglutarate on the level of AGE (a) and TBARS (e) and activity of antioxidant enzymes ((b–d), and (f)) in experimental groups. 1, control group; 2, rats with PIMD; 3, rats with PIMD + Corvitin; 4, rats with PIMD + 2-OG; ^*∗*^*P* < 0.05, ^*∗∗*^*P* < 0.01, and ^*∗∗∗*^*P* < 0.001, compared to control; §*P* < 0.05, §§*P* < 0.01, and §§§*P* < 0.001, compared to PIMD-group.

Our results showed that levels of TBARS and AGEs increased by 2.8 and 1.3 times, respectively, in the plasma of the animals with PIMD compared to the control group (Figures 4(a) and 4(e)), and there is a correlation between these parameters (*r* = 0.61; *P* < 0.05). The use of Corvitin and 2-OG for 6 days instigated reductions in the contents of TBARS and AGEs, but not to the level registered in the control group (Figure 4(e)). It should be noted that the increase of AGEs in the PIMD-group was accompanied by a rise in the glucose level to 6.03 ± 0.63 mmol/l compared to 4.71 ± 0.57 mmol/l in the control group. After the administration of Corvitin, glucose level recovered to the standard value, and with the introduction of 2-OG, it increased further than the level in the PIMD-group (Figure 4(a)).

The combined administration of pituitrin-isoproterenol also led to various changes in the activity of enzymes of the antioxidant system. The most significant differences were observed in enzymes that neutralize hydrogen peroxide. The level of catalase in the blood plasma decreased almost three times (*P* < 0.001; Figure 4(b)), while the activity of glutathione peroxidase in the red blood cells, by contrast, increased 1.5 times (*P* < 0.001; Figure 4(c)). The activity of other enzymes in rats with PIMD changed slightly, with no noticeable difference in comparison to the control group. We only noted the tendency of the glutathione reductase activity to decrease and a slight increase in the SOD level (Figures 4(b) and 4(d)). The content and activity of all studied enzymes increased in animals which received Corvitin in comparison with PIMD-rats, except for SOD whose level was lower than in both the PIMD-rats and control group, while the use of 2-OG reduced the activity of antioxidant enzymes to almost normal values. Interestingly, the relation of glutathione peroxidase activity to those of glutathione reductase (GP/GR index) was two times higher in rats with PIMD, compared to the control group, and did not change after the administration of Corvitin, while the application of 2-OG resulted in a decreased GP/GR index in comparison to the control group (Figure 4(f)).

## 4. Discussion

We investigated the effects of Corvitin and 2-oxoglutarate on the physiology and histology of the heart, behavioral reactions, and markers of carbonyl/oxidative stress in rats with a myocardial damage that was induced by the administration of pituitrin and isoproterenol. Comparing our results with the works of other researchers showed that the chosen scheme of administration of these substances causes changes in the physiological state of animals, in ECG and heart histology, like the clinical manifestations of myocardial infarction of humans [46–49]. The decrease in the glycogen level in the survived cardiomyocytes around the necrotic foci, observed in our study, agrees with the fact that oxygen deprivation due to ischemia stimulates the mobilization of glycogen and the generation of ATP. On the contrary, the presence of reactive inflammatory infiltrates in the necrotic regions contributes to an increase in free radicals, strengthening their negative influence in these areas. Apparently, increased inflammatory infiltration is associated with development of necrotic changes. Therefore, the severity of inflammatory response may indicate the propagation of necrotized areas in the left ventricle [50, 51].

The pituitrin-isoproterenol-induced myocardial damage relates to the development of carbonyl/oxidative stress and imbalance in the antioxidant enzyme system. According to our results, the level of SOD in the erythrocyte hemolysate was virtually unchanged for 6 days after PIMD-induction, possibly because most of the superoxide anion (O^2−^) was already inactivated in the dismutation reactions. Opposite changes in the activities of catalase and glutathione peroxidase, which we noted in the results, may be due to their nonenzymatic glycation [52–54]. Our finding is consistent with the data of other studies, in which the high sensitivity of catalase to nonenzymatic glycation was demonstrated, leading to a decrease in the activity of this enzyme with aging, hyperglycemia, and carbonyl-oxidative stress. Glutathione peroxidase, by contrast, has a low sensitivity to active carbonyl compounds. According to data of Bakala et al. [55], incubation of catalase with 5 mM of fructose for 24 hours resulted in decrease of its activity more than three times, while the activity of GP decreased only on 5% in the similar conditions of the experiment [56]. Furthermore, glutathione peroxidase can reduce the level of AGEs by activating dihydroxyacetone kinase and reducing the dihydroxyacetone content—one of the most active carbonyl compounds [57].

The effects of Corvitin and 2-OG on the activity of the antioxidant enzymes were similar, except for the activity of glutathione-dependent enzymes and their ratio. Under the influence of Corvitin, the increased GP/GR index persisted and was two times higher than usual, while under the action of 2-OG, this coefficient decreased to almost regular values. In our studies, the activity of GP and GR was evaluated in erythrocytes, the metabolism of which is limited due to the absence of cellular organelles, including mitochondria, which produce endogenous 2-OG. Therefore, changes in the activity of antioxidant enzymes under the action of exogenous 2-OG are most likely due to its antioxidative properties. These findings are in agreement with the observations of other scientists [58, 59].

The use of both drugs caused positive dynamics of morphological and functional changes in the damaged myocardium, demonstrating their cardioprotective properties after PIMD. These functions were confirmed by increased cardiomyocyte viability, decreased ischemic injury (such as depression in the ST segment), faster heart rate recovery, increased glycogen content, and reduced myocardial inflammation in animals receiving Corvitin and 2-OG. In addition to the general physiological correction and cardioprotective effect, the drugs also incited an improvement in locomotive and cognitive activities in rats. The neuroprotective effect of quercetin and its derivatives has been discussed previously regarding its antioxidative properties. In our previous studies, an established regulatory action of Corvitin on the metallothionein (MT) and glial fibrillary acidic protein (GFAP) levels in various parts of the brain was shown. It is known that GFAP is responsible for the functional activity of astrocytes and nutrition of neurons, while MT regulates gene expression and cell adaptation to the stress factors [60]. Behavioral activation of rats with PIMD after the treatment by 2-OG is the definitive result of its multifactor impact on brain metabolism. It is well known that 2-OG plays a crucial role in generating energy in nerve cells through the TCA and respiratory chain in the mitochondria. Under conditions of ischemia-reperfusion in PIMD-rats, the activities of the 2-OG dehydrogenase complex, which supplies NADH to the respiratory chain, as well as of the first complex of this chain, are reduced [61, 62]. The diminished actions of these enzymes can be attributed to the low levels of 2-OG used as the primary substrate for the conversion of extra glutamate to glutamine under ischemic conditions [63]. The results of this research serve as proof that exogenous 2-OG in its neuroprotective role decreases the levels of ROS and AGEs by regulating the activity of the antioxidant system while improving energy supply levels by restoring the diminished 2-OG. Thus, the protective effects of Corvitin and 2-OG on myocardial damages, caused by pituitrin-isoproterenol, are provided in many ways, including their robust antioxidative capacity, as established in our investigation.

## 5. Conclusion

Corvitin and exogenous 2-oxoglutarate provide cardio- and neuroprotective protection through their ability to decrease carbonyl/oxidative stress and regulate the activity of the antioxidant system.

## Data Availability

The data used to support the findings of this study are available from the corresponding author upon request.
